# A group decision making method with preference analysis to re-build the Global Entrepreneurship Index

**DOI:** 10.1371/journal.pone.0282993

**Published:** 2023-04-20

**Authors:** Caixiang Chen, Shiliang Mo, Jinling Zhao

**Affiliations:** 1 Logistics and e-Commerce College, Zhejiang Wanli University, Ningbo, China; 2 College of Humanities, Zhejiang Normal University, Jinhua, China; 3 School of Economics, Shen Zhen Polytechnic, Shenzhen, China; 4 Research Center for Finance and Fiscal Rule of Law of The Guangdong-Hong Kong-Mao Greater Bay Area, School of Economics, Shenzhen Polytechnic, Shenzhen, China; 5 Yonyou Technology Research and Development Center, Shenzhen Polytechnic, Shenzhen, China; Universita degli Studi del Molise, ITALY

## Abstract

This study proposes a group decision making (GDM) method with preference analysis to re-build the Global Entrepreneurship Index (GEI). Specifically, a single decision maker is firstly identified using a specified individual judgement about the importance order of three sub-indices of the GEI. A preliminary group decision matrix is constructed in terms of taking all possible individual judgments into account. Then the analysis of the preferential differences and preferential priorities with respect to the preliminary group decision matrix is conducted to obtain a revised group decision matrix, in which preferential differences calculate the weighted differences as the degrees of differences among different alternatives for each decision maker, preferential priorities describe the favorite ranking of alternatives for each decision maker. Finally, we employ the Stochastic Multicriteria Acceptability Analysis for group decision-making (SMAA-2) to create the holistic acceptability indices for measuring the entrepreneurship performance. In addition, a satisfaction index is developed to indicate the merits of proposed GDM method. A case study using the GEI-2019 of 19 G20 countries is carried out to validate our GDM method.

## 1. Introduction

Entrepreneurship has come to be perceived as an engine of social and economic development throughout the world. Entrepreneurship is in particular defined as “*the dynamic*, *institutionally embedded interaction between entrepreneurial attitudes*, *entrepreneurial abilities*, *and entrepreneurial aspirations by individuals*, *which drives the allocation of resources through the creation and operation of new ventures*” [1, 2]. This definition of entrepreneurship not only is driven by opportunity, but also regards the level of technology. The empirical evidence has indicated that entrepreneurial activity varies across stages of economic development, with a U-shaped relationship between the rate of entrepreneurship and level of development [1]. Similarly, Du et al. explore the impact of entrepreneurship on national economic efficiency and find that more entrepreneurship does not always benefit economic growth [3].

The Global Entrepreneurship and Development Institute (GEDI Institute) is the world’s leading research institution dedicated to understanding the relationship between entrepreneurship, economic development, and prosperity, using the data provided by the Global Entrepreneurship Monitor (GEM) and the World Economic Forum (WEF) Global Competitiveness Index (GCI). Known as a breakthrough in evaluating the quality and dynamics of entrepreneurship ecosystems at a national and regional level since 2009, the Global Entrepreneurship Index (GEI) is composed of three sub-indices, namely, 3As: entrepreneurial attitudes (ATT), entrepreneurial abilities (ABT), and entrepreneurial aspirations (ASP). 3As stand on 14 pillars, each of which contains an individual and an institutional variable that correspond to the micro- and the macro-level aspects of entrepreneurship. Entrepreneurial attitudes are societies’ attitudes toward entrepreneurship, which are defined as a population’s general feelings about recognizing opportunities, knowing entrepreneurs personally, endowing entrepreneurs with high status, accepting the risks associated with business startups, and having the skills to launch a business successfully. Entrepreneurial abilities refer to the entrepreneurs’ characteristics and those of their businesses. Entrepreneurial aspirations reflect the quality aspects of startups and new businesses. The GEI framework has been extensively validated by rigorous academic review and widely reported in media, including Financial Times, The Economist, Forbes, and The Wall Street Journal. Table 1 below reports the structure of GEI-2019. The arithmetical average of the scores attained on ATT, ABT and ASP is recognized as the comprehensive score of the GEI:

$$GEI=\frac{ATT+ABT+ASP}{3}$$

**Table 1:** The structure of GEI-2019.

	Sub-indices	Pillars	Variables (individual / institutional)
GEI	Attitudes	Opportunity Perception	Opportunity recognition
			Freedom (Economic freedom × Property rights)
		Startup skills	Skill perception
			Education (Tertiary education × Quality of education)
		Risk acceptance	Risk perception
			Country risk
		Networking	Know entrepreneurs
			Agglomeration (urbanization × infrastructure)
		Culture support	Career status
			Corruption
	Abilities	Opportunity startup	Opportunity motivation
			Governance (taxation × good governance)
		Technology absorption	Technology level
			Technology absorption
		Human capital	Education level
			Labor market (staff training × labor freedom)
		Competition	Competitors
			Competitiveness (market dominance × regulation)
	Aspiration	Product innovation	New product
			Tech transfer
		Process innovation	New technology
			Science (Gerd × ((average quality of scientifical institutions + availability of scientists and engineers))
		High growth	Gazelle
			Finance and strategy (venture capital × business sophistication)
		Internationalization	Export
			Economic complexity
		Risk capital	Informal investment
			Depth of capital market

Although the equal-weighting aggregation scheme is simple-to-implement and easy-to-understand, its effectiveness is highly constrained by many remarkable bottlenecks [4, 5]:

1. Performance may not be improved because the connection with the weights is lost.
2. This is not Pareto optimal. As a result, it would be difficult to accept the results of all alternatives.
3. There is a possibility of compensation. The lower-scored sub-indices can be compensated by higher-scored sub-indices.

Moreover, from actual experience, different countries/regions may have different intrinsic individual judgments about the order of importance of sub-indices, ensuring that each country/region chooses the one that best suits itself. This motivates a methodological improvement to encompass different individual preferences in measuring and comparing entrepreneurship performance based on the GEI.

This research proposes a group decision-making (GDM) method to re-build the GEI published by the GEDI, for better measuring the national/regional entrepreneurship performance. It is not uncommon for the above individual judgments to show considerable variability. This happens naturally in the context of management decision-making and social election [6]. Therefore, we consider all possible individual judgments about the importance order among ATT, ABT, and ASP. Certain importance order can be directly defined as an individual decision maker. This is reasonable because personal judgments are generally different between decision makers and decision makers [5]. By a sophisticated mathematical transformation, the optimal assessment result of each decision maker is derived as a closed-form formulation. In the presence of multiple decision makers, we obtain a preliminary group decision matrix and thus explore its preference structures. More specifically, we study preferential difference and priorities for the evaluation of alternatives by individual decision makers in a decision group, in which preferential differences calculate the weighted differences as the degrees of differences among different alternatives for each decision maker, preferential priorities describe the favorite ranking of alternatives for each decision maker [7–9]. We modify the preliminary group decision matrix considering preferential differences and preferential priorities, and thereby make the use of Stochastic Multicriteria Acceptability Analysis for group decision-making (SMAA-2) to create a set of holistic acceptability indices for rebuilding the GEI [10, 11].

The main purpose of this study is to re-design the GEI through the development of a GDM method with preference analysis. Compared to the existing literature innovating the methods of measuring entrepreneurial performance, our work contributes to this growing topic in the following aspects:

1. This article identifies decision makers using the individual judgments about the importance order among ATT, ABT, and ASP, and builds a preliminary group decision matrix by considering all possible individual judgments.
2. This article analyzes preferential differences and preferential priorities for alternative evaluation by individual decision makers, according to which a modified group decision matrix is proposed.
3. This article implements the group decision aggregation using SMAA-2 and develops a satisfaction index to demonstrate the superiority of our method.

The rest of this paper is organized as follows. Section 2 describes the research problem. Related studies are reviewed in Section 3. The method is proposed in Section 4. An empirical study for a panel of 19 G20 countries is carried out in Section 5. We conclude in Section 6 by discussing the details of our method and suggestions for future research.

## 2. Literature reviews

### 2.1 Entrepreneurship performance measurement

This study is most relevant to the literature on developing methods to measure entrepreneurship performance. Marcotte reviews and analyzes the existing entrepreneurship indices associated with their conceptual and methodological dimensions, which are further compared in 21 OECD countries [12]. Bonyadi and Sarreshtehdari conduct a critical review to assess the existing shortfalls in the model used for the computation of the GEI, especially for economies that have not participated in the GEM annual surveys [13]. Zoltán J. Ács et al. provide a comprehensive comparison between the GEM dataset and the World Bank Group Entrepreneurship Survey (WBGES) dataset designed to measure entrepreneurship [14]. Zoltán J. Ács et al. propose a novel concept of National Systems of Entrepreneurship and provide an approach to characterizing them [15]. Zoltán J. Ács et al. develop a regional application of the GEI that captures the contextual features of entrepreneurship across regions in Spain, with the identification of weaknesses in the incentive structure that affect regional development [16]. Ibrahim et al. construct the Entrepreneurship Index as a measurement tool that effectively determines if one possesses the prospect of becoming a successful entrepreneur by assessing several essential aspects [17]. Song et al. propose a holistic acceptability global entrepreneurship index, ignoring the consideration preference structures [18]. Mas-Tur et al. employ a configurable approach to disentangling the relationship between entrepreneurship and sustainable development [19]. Apart from these, Edmundo Inácio Júnior et al. claim that the Key Performance Indicators’ analysis leads to a misinterpretation of the dynamics of National Systems of Entrepreneurship (NSEs), based on an efficiency analysis of the GEI [20].

### 2.2 GDM methods and applications

This work is also closely related to the literature on GDM. Keeney proposes a general normative model of group decision analysis based on a set of logical and operational assumptions similar to those used for individual decision analysis [21]. Melkonyan and Safra examine the random preference model and the possibility of violations of weak stochastic transitivity for the models with expected utility and betweenness like preferences [6]. Liang et al. develop a prospect theory-based method to fuse the individual preference approval structures in GDM [22]. Dong et al. provide a trust relationships consensus reaching process (CRP) with a feedback mechanism including two approaches of facilitating consensus reaching: 1) the leader-based preference adjustment and 2) the trust relationships improvement [23]. Xu et al propose a new method to check and improve the consistency of individual intuitionistic fuzzy preference relations and consensus among experts [24]. Wan et al. investigate a GDM method based on additive consistent interval-valued Atanassov intuitionistic fuzzy preference relations and likelihood comparison algorithm [25]. Wan et al. explore another GDM method that considers group consensus and multiplicative consistency of interval-valued intuitionistic fuzzy preference relations [26]. Wan et al. give a novel two-stage consensus reaching process (CRP) for MCGDM with linguistic intuitionistic fuzzy variables considering experts’ willingness of modifying preference information [27]. Wan et al. provide a new personalized individual semantic (PIS) based CRP for large-scale group decision making (LSGDM) with probabilistic linguistic preference relations and applies to the selection of COVID-19 surveillance plans [28]. Tang and Liao summarize the challenges from conventional group decision making to LSGDM and present a state-of-the-art survey of main achievements in this field [29]. In addition to these theoretical innovations in GDM, there are many applications of GDM in different areas, for example, credit scoring [30], emergency decision support [31], e-procurement service provider selection Ramkumar and Jenamani [32], industrial robot selection [33], energy performance comparison [5], determining passenger demands and evaluating passenger satisfaction [34], sustainable building material selections [35], site selection of high-speed railway station [36], and among others.

## 3. Problem description

Released by The Global Entrepreneurship and Development Institute (The GEDI Institute), the GEI is a composite indicator that measures the quality and dynamics of entrepreneurship ecosystems at a national and regional level across the 3As: entrepreneurial attitudes, entrepreneurial abilities, and entrepreneurial aspirations. The entrepreneurship performance of nation/region *i*, *i* = 1, 2, …, *m* associate with sub-index *j*, *j* = 1, 2, 3 is represented by *x*_*ij*_, the value of which is usually normalized to mitigate adverse effects of data size using various regularization formulas, such as mean regularization, mix-max regularization, and Z-score regularization. In reality, trade-off justification through aggregation of equal weights is widely used. However, there are two-fold drawbacks with respect to the equal weighting scheme. First, this plausible decision completely ignores the fact that different decision makers may have different individual judgments about the importance of these sub-indices. Second, the common practice for an individual decision maker is to determine a set of weights with respect to each sub-index *w*_*j*_, *j* = 1, 2, 3, in a subjective, or objective, or subjective-objective integrated manner. Determining weights actually play an important role in these multi-attribute performance evaluation problems. Appropriate weight elicitation methods can significantly increase the efficiency of the final decision. Numerous methods have been developed with significant advantages to obtain optimal weights. However, reaching consensus on exact weights even when the ranking is known remains a controversial issue. Therefore, it is extremely significant to comprehensively consider the wisdom of multiple decision makers. To the best of our knowledge, we are the first to re-build the GEI in such a GDM framework.

## 4. Methodology

We develop a GDM method with preference analysis for the general case in this section, which can be easily employed to re-build the GEI with three sub-indices, namely, Attitudes, Abilities and Aspiration. Consider the general situation in which the performance of entity *i*, *i* = 1, 2, …, *m* is calculated in terms of taking the arithmetic average of the scores attained on *n* sub-indices, *x*_*ij*_, *i* = 1, 2, …, *m*, *j* = 1, 2, …, *n*, that is,

$$S_{i}=\frac{x_{i1}+x_{i2}+\ldots +x_{in}}{n},\;i=1,2,\ldots ,m$$ (1)

in which *x*_*ij*_ are standardized data that can effectively alleviate the adverse impact of various data magnitudes. The consideration of all possible importance sequence of sub-indices could deal with the drawbacks associated with the arithmetic average scheme [5].

The proposed GDM method consists of three phases. First, a preliminary group decision making matrix is formulated, in which a single decision maker is identified by an importance sequence of all sub-indices. Second, both the preference levels and preference priorities among entities for individual decision makers are analyzed to derive a revised group decision making matrix. Third, the SMAA-2 is used to implement the group decision aggregation.

### 4.1 Preliminary group decision making matrix

In line with Fu et al. [5], there are *n*! individual decision makers identified by extensively considering the importance orders among the sub-indices. For the ease of demonstration, we only investigate the scenario with *w*_1_≥*w*_2_≥…≥*w*_*n*_, and *w*_*j*_, *j* = 1, 2, …, *n* is the importance degree of sub-index *j*. This scenario is reasonably identified as an individual decision maker and the decision results could be easily migrated to other decision makers. In this manner, the performance result for entity *i* can be determined by solving the following linear program:

$$\begin{array}{l} v_{i}^{1}=\text{max}\;\sum_{j=1}^{n}x_{ij}w_{j}\\ \begin{array}{cc} s.t. & w_{1}\geq \end{array}w_{2}\geq \ldots \geq w_{n}\\ \begin{array}{cc} & \sum_{j=1}^{n}w_{j}=1, \end{array}w_{j}\geq 0 \end{array}$$ (2)

For the auxiliary parameters *α*_*j*_ ≥ 0, *j* = 1, 2, …, *n*, the ranked weights are defined as $w_{k}=\sum_{j=k}^{n}\alpha_{j}$. This is consistent with given individual preference among sub-indices, *w*_1_≥*w*_2_≥…≥*w*_*n*_. Let *β*_j_ = *jα*_*j*_,

$$\sum_{j=1}^{n}\beta_{j}=\sum_{j=1}^{n}j\alpha_{j}=\sum_{j=1}^{m}\sum_{k=j}^{n}\alpha_{k}=\sum_{j=1}^{n}w_{j}=1$$ (3)

Moreover, we define $s_{ik}=\frac{1}{k}\sum_{j=1}^{k}x_{ij}$, *k* = 1, 2, …, *n*, then

$$\sum_{j=1}^{n}x_{ij}w_{j}=\sum_{j=1}^{m}\sum_{k=j}^{n}x_{ij}\alpha_{k}=\sum_{j=1}^{m}\sum_{k=j}^{n}x_{ij}\left(\frac{\beta_{k}}{k}\right)=\sum_{k=1}^{n}\beta_{k}\left(\frac{1}{k}\sum_{j=1}^{k}x_{ij}\right)=\sum_{k=1}^{n}\beta_{k}s_{ik}$$ (4)

The linear program (2) therefore equals to the model below:

$$\begin{array}{l} v_{i}^{1}=\text{max}\;\sum_{k=1}^{n}\beta_{k}s_{ik}\\ \begin{array}{cc} s.t. & \begin{array}{cc} \sum_{k=1}^{n}\beta_{k}=1, & \beta_{k}\geq 0 \end{array} \end{array} \end{array}$$ (5)

Let $\overset{⌢}{k}\in \left\{1,2,\ldots ,n\right\}$ satisfies that $s_{i\overset{⌢}{k}}=\text{max}\left\{s_{ik}\right\}$, the optimal solution to linear program (5) is then derived as

$$\beta_{k}=\left\{\begin{array}{cc} 1, & k=\overset{⌢}{k}\\ 0, & otherwise \end{array}\right.$$ (6)

As a consequence, the performance result of entity *i* with *w*_1_≥*w*_2_≥…≥*w*_*n*_ could be easily derived as the closed form: $v_{i}^{1}=\underset{k}{\text{max}}\left\{s_{ik}\right\}=\underset{k}{\text{max}}\left\{\frac{1}{k}\sum_{j=1}^{k}x_{ij}\right\}$, *k* = 1, 2, …, *n*. This scheme is simple-to-implement and easy-to-understand, and could be readily migrated to other situations. The whole procedure does not require using any linear optimizer and is implemented on the spreadsheet package without determining the precise values of weights.

In the consideration of all possible importance sequence of sub-indices, we construct a preliminary group decision matrix with *n*! individual decision makers:

$$V_{m\times n}=\left[\begin{array}{ccccc} v_{1}^{1} & \cdots & v_{1}^{p} & \cdots & v_{1}^{n!}\\ v_{2}^{1} & \cdots & v_{2}^{p} & \cdots & v_{2}^{n!}\\ \vdots & \vdots & \vdots & \vdots & \vdots \\ v_{m}^{1} & \cdots & v_{m}^{p} & \cdots & v_{m}^{n!} \end{array}\right]$$ (7)

where $v_{i}^{p}$, *i* = 1, 2, …, *m*, *p* = 1,2,…, *n*! denotes the score of entity *i* evaluated by individual decision maker *p*. The preference ranking of alternative *i* by decision maker *p*, is therefore determined by the $v_{i}^{p},i=1,2,\ldots ,m,p=1,2,\ldots ,n!$. However, the group decision aggregation using the preliminary group decision matrix is intuitive and may lack the investigation of the preference levels and preference priorities among entities for individual decision makers [5, 7].

### 4.2 Revised group decision making matrix

In response to the aforementioned shortcomings associated with the preliminary group decision matrix, this section untangles and integrates the preferential differences and preferential priorities to capture the weight differences and alternative priorities for better aggregation of individual judgments with a collective commitment. Specifically, preferential differences calculate the weighted differences as the degrees of differences among different alternatives for each decision maker, preferential priorities describe the favorite ranking of alternatives for each decision maker.

The previous studies have claimed that each decision maker may have her/his own preferential differences among alternatives, which have been measured by means of developing several meaningful indices [7–9]. In accordance with Huang and Li [7], the weighted difference between alternatives i and t by decision maker p is defined as

$$\begin{array}{ccc} \alpha_{pit}=\left|v_{i}^{p}-v_{t}^{p}\right|, & 0\leq \alpha_{pit}\leq 1, & i\neq t \end{array}$$ (8)

in which $v_{i}^{p}$ denote the performance result of entity *i* evaluated by individual decision maker *p*. Since *α*_*pit*_ only indicate the preferential differences for decision maker *p*, we need to normalize the mean differences for the n! decision makers to derive the preferential differences in the decision group. As a result, the weighted difference for decision maker p is obtained as

$$\begin{array}{cccc} \alpha_{p}=\frac{\sum_{i<t}\alpha_{pit}}{\sum_{p=1}^{n!}\sum_{i<t}\alpha_{pit}}, & 0<\alpha_{p}<1, & i\neq t, & 1\leq i<t\leq m \end{array}$$ (9)

in which *α*_*p*_ is computed by taking the sum of *α*_*pit*_ for $\frac{m\left(m-1\right)}{2}$ times to consider all possible combinations of alternative pairs.

A small *α*_*p*_ indicates that the preferential difference for decision maker p is small. This implies that the decision maker *p* is unable to or has no interest in decisively discriminating these alternatives, and may have similar preferences for all alternatives. In this sense, the weighted differences could be utilized as a means for an individual decision maker to highlight her/his preferential differences in the decision group.

In addition, decision makers would be in particular aware of whether their most preferred alternative is adopted in a group decision problem, and thus assign more importance to this alternative than to others [7–9]. In this regard, the preferential priority of alternative *i* by decision maker *p* is defined as $\beta_{pi}=\frac{m}{\varphi_{pi}}$, in which *φ*_*pi*_ represents the preference ranking of alternative *i* by decision maker *p*. In the presence of tied alternatives with the identical ranking, they would be assigned with the ranking that is the average of their original ranking positions. Then the preferential priorities of alternative i are aggregated as $\beta_{i}=\sum_{p=1}^{n!}\beta_{pi}$, and would be normalized to obtain the preferential priority of alternative *i* in the decision group as

$$\beta_{i}^{G}=\frac{\beta_{i}}{\sum_{i=1}^{m}\beta_{i}}$$ (10)

Obviously, the preferential priority particularly gives more weight to the best ranked alternative, and thus is realistic and pragmatic for decision makers because it is able to indicate the extent to which decision makers expect their most favorite alternative to be adopted in a group decision problem.

In summary, based on the above preferential difference and preferential priority analysis, a revised group decision matrix is obtained below to make the consensus- and commitment- achieving in a decision group more realistic and reasonable.


$$V_{m\times n!}^{*}=\left[\begin{array}{ccccc} v_{1}^{1}\times \alpha_{1}\times \beta_{1}^{G} & \cdots & v_{1}^{p}\times \alpha_{p}\times \beta_{1}^{G} & \cdots & v_{1}^{n!}\times \alpha_{n!}\times \beta_{1}^{G}\\ v_{2}^{1}\times \alpha_{1}\times \beta_{2}^{G} & \cdots & v_{2}^{p}\times \alpha_{p}\times \beta_{2}^{G} & \cdots & v_{2}^{n!}\times \alpha_{n!}\times \beta_{2}^{G}\\ \vdots & \vdots & \vdots & \vdots & \vdots \\ v_{m}^{1}\times \alpha_{1}\times \beta_{m}^{G} & \cdots & v_{m}^{p}\times \alpha_{p}\times \beta_{m}^{G} & \cdots & v_{m}^{n!}\times \alpha_{n!}\times \beta_{m}^{G} \end{array}\right]$$ (11)

### 4.3 Group decision aggregation using SMAA-2

As for the revised group decision matrix $V_{m\times n!}^{*}$, this study proposes to employ SMAA-2 to aggregate alternative-wide performance. Relative to Stochastic Multicriteria Acceptability Analysis (SMAA), decision makers do not have to state their preferences explicitly or implicitly. SMAA examines the weight space and describes the score that puts each option in the preferred ranking position [11]. The rank acceptability indices are developed to measure the variety of difference preferences that support each alternative the best rank. SMAA-2 extends the analysis to the sets of weight vectors for any rank from best to worst for each decision alternative [10]. Finally, a set of holistic acceptability indices are formulated by integrating the rank acceptabilities using metaweights.

#### 4.3.1 Preliminaries

We examine a common GDM scenario with *m* alternatives that are assessed by *n*! group members, where neither the assessment values of group members nor the corresponding weights are precisely acknowledged, and a decision maker is responsible for consolidating the group decision. It is further assumed that the preference characteristic of the decision maker is expressed by a real-valued utility function *u*(*x*_*i*_, *w*), where a weight vector *w* quantifies the subjective preference of that decision maker. Imprecise or uncertain attribute values are indicated by random variables *ξ*_*is*_, using implicit or estimated joint probability distribution and density functions *f*(*ξ*) in the space *X* ∈ *R*^*m*×*n*!^. Furthermore, decision maker’s unknown or partially known preferences are represented by a weight distribution with density function *f*(w) in the set of feasible weights *W*, which is defined as

$$W=\left\{w\in R^{n!}:w\geq 0\;and\;\sum_{s=1}^{n!}w_{s}=1\right\}$$

Complete lack of knowledge about weights is represented in a “Bayesian” spirit by a uniform weight distribution in *W*. The distribution has the density function $f\left(w\right)=\frac{1}{vol\left(W\right)}$, in which (*n*−1)-dimensional volume of the feasible weight simples is $vol\left(W\right)=\frac{\sqrt{n!}}{\left(n!-1\right)!}$ [10]. Then utility function is utilized to map stochastic criteria values and weight distributions into utility distributions *u*(*ξ*_*i*_, *w*).

As a result, SMAA determines for each alternative the set of favorable weights *W*_*i*_(*ξ*) as:

$$W_{i}\left(\xi \right)=\left\{w\in W:u\left(\xi_{i},w\right)\geq u\left(\xi_{k},w\right),\forall k\right\}$$

Any weight *w*∈*W*_*i*_(*ξ*) makes the utility of *x*_*i*_ no less than the utility of any other alternatives.

We develop the following ranking function to define the rank of each alternative as an integer from the best rank (= 1) to the worst rank (= m):

$$rank\left(\xi_{i},w\right)=1+\sum_{k}\rho \left(u\left(\xi_{k},w\right)>u\left(\xi_{i},w\right)\right)$$ (12)

in which *ρ* (false) = 0 and *ρ* (true) = 1. SMAA-2 is proposed based on analyzing the sets of favorable rank weight $W_{i}^{r}\left(\xi \right)$, which are defined as:

$$W_{i}^{r}\left(\xi \right)=\left\{w\in W:rank\left(\xi_{i},w\right)=r\right\}$$ (13)

A weight $w\in W_{i}^{r}\left(\xi \right)$ assigns utilities for the alternatives in this manner so that alternative *x*_*i*_ obtains rank *r*.

#### 4.3.2 Meaningful measures

The first measure is the rank acceptability index $b_{i}^{r}$, which evaluates the variety of different valuations granting alternative *x*_*i*_ rank *r*. The rank acceptability index is calculated as a multidimensional integral over the criteria distribution and the favorable rank weights using

$$b_{i}^{r}=\int_{x}f\left(\xi \right)\int_{W_{i}^{r}\left(\xi \right)}f\left(w\right)dwd\xi$$ (14)

The rank acceptability indices can be illustrated graphically to investigate how different varieties of weights support each rank for each alternative. Obviously, the rank acceptability indices distribute in the range [0, 1]. $b_{i}^{r}=1$ implies that the alternative *x*_*i*_ always achieve the rank *r*, no matter the choice of weights, and $b_{i}^{r}=0$ means that the alternative *x*_*i*_ never obtain the rank *r*, irrespective to the determination of weights as well.

The weight space corresponding to the k best ranks (*kbr*) for an alternative can be described by means of the central *kbr* weight vector $w_{i}^{k}$, which is computed by

$$w_{i}^{k}=\frac{\int_{X}f\left(\xi \right)\sum_{r=1}^{k}\int_{W_{i}^{r}\left(\xi \right)}f\left(w\right)dwd\xi }{\sum_{r=1}^{k}b_{i}^{k}}$$ (15)

The *kbr* confidence factor $p_{i}^{k}$ is defined as the probability that the alternative receives any rank from 1 to *k* if the central *kbr* weight vector is selected, and is calculated as an integral over the criteria distribution in *X* by

$$p_{i}^{k}=\int_{\xi :rank\left(\xi_{i},w_{i}^{k}\right)\leq k}f\left(\xi \right)d\xi$$ (16)

#### 4.3.3 Holistic acceptability index

The problem of comparing the alternatives in terms of their rank acceptabilities is recognized as a “second-order” multicriteria decision problem [10]. This motivates the development of a complementary approach combining the rank acceptabilities to formulate holistic acceptability indices ah for each alternative:

$$a_{i}^{r}=\sum_{r=1}^{m}\theta^{r}b_{i}^{r}$$ (17)

where *θ*^*r*^ are so-called metaweights, satisfying 0 ≤ *θ*^*m*^ ≤ *θ*^*m*−1^ ≤…≤ *θ*^2^ ≤ *θ*^1^ ≤ 1.

Consequently, on the strength of the revised group decision matrix taking into account both preferential differences and preferential priorities, the SMAA-2 method is used to build the holistic acceptability indices for each alternative, which could be employed to implement the performance evaluation and comparison of all alternatives.

## 5. An empirical study

To validate our GDM method, an empirical study is conducted to re-build the Global Entrepreneurship Index using the data of GEI-2019, which reports the rankings of 137 countries/regions, and provides confidence intervals for the GEI. Nineteen individual G20 countries are selected to illustrate the GDM method. The G20 consists of 19 individual countries and the European Union (EU). The EU is represented by the European Commission and the European Central Bank. Together, the G20 countries account for about 90% of global gross domestic product (GWP), 80% of world trade (or 75% if not traded within the EU), two-thirds of the world’s population, and about half of the world’s land area. The ATT, ABT, ASP and GEI raw data of the 19 individual economics of G20 are given in the Table 2 below, in which the countries are alphabetically listed.

**Table 2:** The GEI-2019 data for 19 G20 countries.

Country	ATT	ABT	ASP	GEI	Normalized ATT	Normalized ABT	Normalized ASP
Argentina	27.9	24.4	25.7	26.0	0.1215	0.0997	0.1167
Australia	80.1	65.2	74.1	73.1	0.3487	0.2664	0.3365
Brazil	15.6	8.3	24.5	16.1	0.0679	0.0339	0.1113
Canada	83.8	79.4	78.0	80.4	0.3648	0.3244	0.3543
China	34.2	66.6	36.8	45.9	0.1489	0.2721	0.1671
France	66.8	77.7	56.8	67.1	0.2908	0.3174	0.2580
Germany	68.2	74.0	57.8	66.7	0.2969	0.3023	0.2625
India	23.6	28.9	22.7	25.1	0.1027	0.1181	0.1031
Indonesia	28.4	17.2	32.3	26.0	0.1236	0.0703	0.1467
Italy	40.5	57.0	37.9	45.1	0.1763	0.2329	0.1721
Japan	61.4	67.1	31.4	53.3	0.2673	0.2741	0.1426
Mexico	25.0	25.9	30.4	27.1	0.1088	0.1058	0.1381
Russia	27.6	19.6	27.0	24.7	0.1202	0.0801	0.1226
Saudi Arabia	29.8	39.6	56.8	42.1	0.1297	0.1618	0.2580
South Africa	29.3	39.2	26.3	31.6	0.1276	0.1602	0.1194
South Korea	46.3	60.1	67.8	58.1	0.2016	0.2455	0.3079
Turkey	33.2	51.6	34.6	39.8	0.1445	0.2108	0.1571
United Kingdom	82.6	76.3	73.5	77.5	0.3596	0.3117	0.3338
United States	89.7	87.2	83.5	86.8	0.3905	0.3563	0.3792

In view of that the GEI is composed of ATT, ABT and ASP, we reasonably identify six ($A_{3}^{3}=6$) decision makers using six individual judgments about the importance sequence of them. That are, Decision Maker TBS:ATT ≻ ABT ≻ ASP; Decision Maker TSB:ATT ≻ ASP ≻ ABT; Decision Maker BTS: ABT ≻ ATT ≻ ASP; Decision Maker BST: ABT ≻ ASP ≻ ATT; Decision Maker STB: ASP ≻ ATT ≻ ABT; Decision Maker SBT: ASP ≻ ABT ≻ ATT. For each individual decision maker, the optimal decision results associated with each country could be derived as a closed-form expression, without the elicitation of exact weights of ATT, ABT and ASP, which are given in the following Table 3. Moreover, Table 3 also reports the preferential differences *α*_*p*_ and preferential priorities $\beta_{i}^{G}$. It is observed that decision makers TBS and SBT has the largest and smallest preferential differences as *α*_*TBS*_ = 0.1746 and *α*_*SBT*_ = 0.1585. This indicates that decision maker TBS has the largest interest in decisively discriminating these countries, and decision maker SBT has similar preference among these countries. In addition, the preferential priority of United States is largest ($\beta_{\text{United}\;\text{States}}^{G}=0.2819$). This implies that United States is most preferred for the group in terms of ranking. It is an intuitive decision result since all six decision makers put United States first on the table.

**Table 3:** The preliminary group decision matrix.

Country	TBS	TSB	BTS	BST	STB	SBT	$\beta_{i}^{G}$
Argentina	0.1215	0.1215	0.1126	0.1126	0.1191	0.1167	0.0171
Australia	0.3487	0.3487	0.3172	0.3172	0.3426	0.3365	0.0697
Brazil	0.0710	0.0896	0.0710	0.0726	0.1113	0.1113	0.0151
Canada	0.3648	0.3648	0.3478	0.3478	0.3595	0.3543	0.1409
China	0.2105	0.1960	0.2721	0.2721	0.1960	0.2196	0.0309
France	0.3041	0.2908	0.3174	0.3174	0.2887	0.2887	0.0564
Germany	0.2996	0.2969	0.3023	0.3023	0.2872	0.2872	0.0463
India	0.1104	0.1080	0.1181	0.1181	0.1080	0.1106	0.0167
Indonesia	0.1236	0.1352	0.1135	0.1135	0.1467	0.1467	0.0198
Italy	0.2046	0.1938	0.2329	0.2329	0.1938	0.2025	0.0269
Japan	0.2707	0.2673	0.2741	0.2741	0.2280	0.2280	0.0364
Mexico	0.1176	0.1235	0.1176	0.1219	0.1381	0.1381	0.0189
Russia	0.1202	0.1214	0.1076	0.1076	0.1226	0.1226	0.0168
Saudi Arabia	0.1832	0.1939	0.1832	0.2099	0.2580	0.2580	0.0285
South Africa	0.1439	0.1357	0.1602	0.1602	0.1357	0.1398	0.0209
South Korea	0.2517	0.2547	0.2517	0.2767	0.3079	0.3079	0.0425
Turkey	0.1777	0.1708	0.2108	0.2108	0.1708	0.1840	0.0242
United Kingdom	0.3596	0.3596	0.3357	0.3350	0.3467	0.3350	0.0900
United States	0.3905	0.3905	0.3753	0.3753	0.3849	0.3792	0.2819
α_p_	0.1746	0.1694	0.1676	0.1666	0.1632	0.1585	

Based upon the preferential differences *α*_*p*_ and preferential priorities $\beta_{i}^{G}$, the revised group decision matrix is obtained according to (11) and presented in Table 4 below. It is evident that all values have been modified to better reveal the preference structure of the group decision matrix.

**Table 4:** Revised group decision matrix.

Country	TBS	TSB	BTS	BST	STB	SBT
Argentina	0.000363	0.000352	0.000323	0.000321	0.000333	0.000317
Australia	0.004243	0.004117	0.003705	0.003683	0.003896	0.003718
Brazil	0.000187	0.000229	0.000180	0.000183	0.000274	0.000267
Canada	0.008978	0.008712	0.008215	0.008167	0.008269	0.007916
China	0.001137	0.001027	0.001410	0.001402	0.000989	0.001077
France	0.002994	0.002778	0.002999	0.002981	0.002656	0.002581
Germany	0.002423	0.002330	0.002346	0.002332	0.002171	0.002109
India	0.000321	0.000305	0.000330	0.000328	0.000293	0.000292
Indonesia	0.000428	0.000454	0.000377	0.000375	0.000474	0.000461
Italy	0.000961	0.000883	0.001050	0.001044	0.000851	0.000864
Japan	0.001723	0.001651	0.001674	0.001665	0.001356	0.001318
Mexico	0.000387	0.000395	0.000372	0.000383	0.000425	0.000413
Russia	0.000352	0.000345	0.000303	0.000301	0.000336	0.000326
Saudi Arabia	0.000913	0.000938	0.000876	0.000998	0.001202	0.001167
South Africa	0.000526	0.000482	0.000562	0.000559	0.000464	0.000464
South Korea	0.001866	0.001833	0.001791	0.001958	0.002134	0.002073
Turkey	0.000751	0.000701	0.000855	0.000850	0.000675	0.000706
United Kingdom	0.005654	0.005486	0.005065	0.005026	0.005095	0.004783
United States	0.019220	0.018650	0.017730	0.017626	0.017704	0.016948

**Fig 1:**
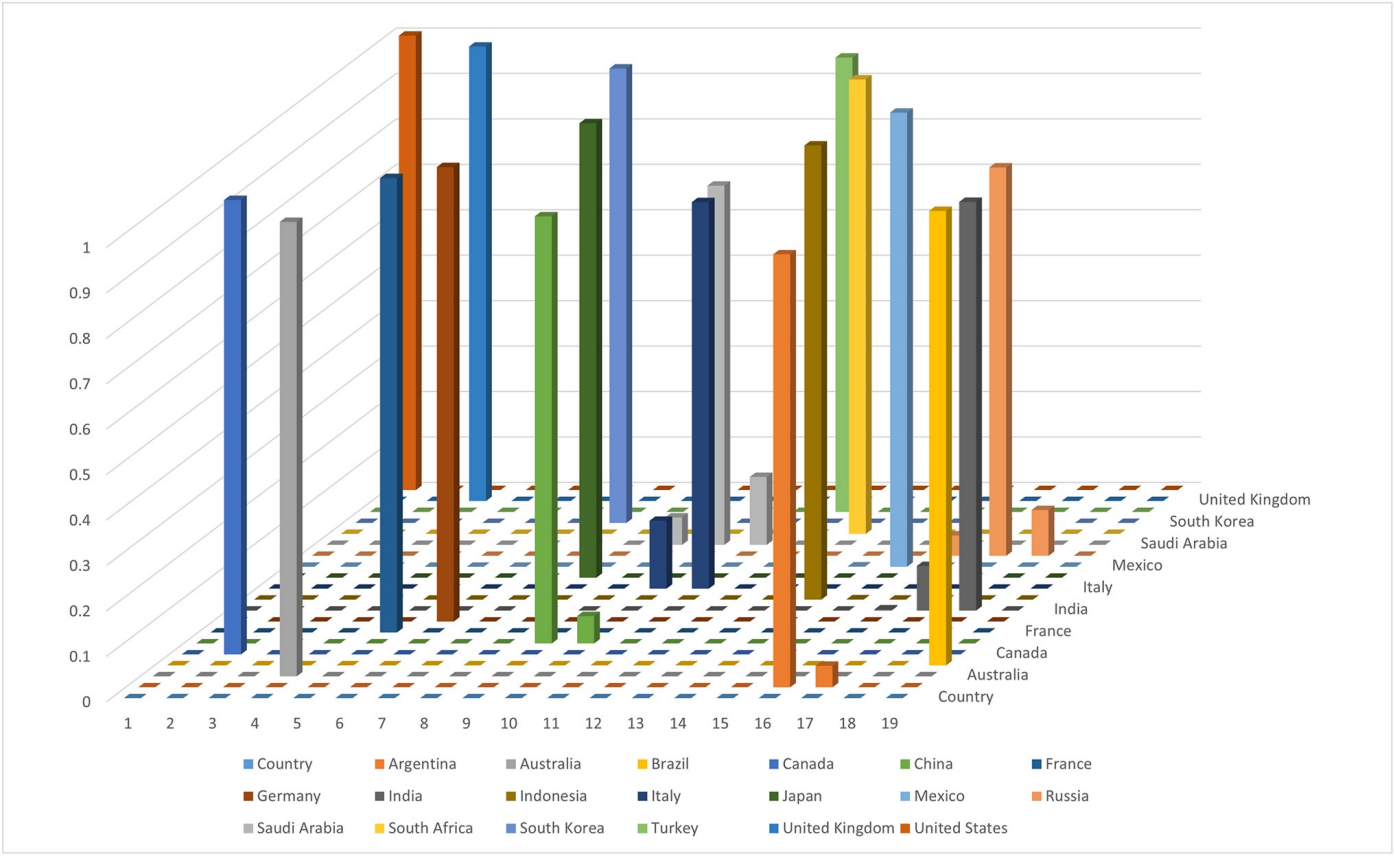
Ranking comparisons.

In what follows, we apply the SMAA-2 to implement the group decision aggregation for the revised group decision matrix as Table 4. The rank acceptability levels can be effectively obtained using open-source software developed by Tervonen [37]. As for the decision with ranked weights, Barron and Barrett claim that ranked-order centroid (ROC) is more straightforward, accurate and efficacious, and is able to provide a reasonable implementation instrument [38]. The ROC is thus used to derive the metaweights for building the holistic acceptability indices: $\alpha^{r}\left(ROC\right)=\frac{1}{19}\sum_{s=r}^{19}\frac{1}{s}$, *r* = 1, 2, …, 19. Finally, the rank acceptability levels and holistic acceptability indices are reported in the following Table 5 and Fig 1. We observe that the rankings of Argentina, Australia, Brazil, Canada, France, Germany, Japan, South Africa, South Korea, Turkey, United Kingdom, and United States are unchanged, while other countries have multiple possibilities to be ranked at different ranking positions.

**Table 5:** Rank acceptability index and holistic acceptability index.

Country	b^1^	b^2^	b^3^	b^4^	b^5^	b^6^	b^7^	b^8^	b^9^	b^10^	b^11^	b^12^	b^13^	b^14^	b^15^	b^16^	b^17^	b^18^	b^19^	$a_{i}^{h}$
Argentina	0.0000	0.0000	0.0000	0.0000	0.0000	0.0000	0.0000	0.0000	0.0000	0.0000	0.0000	0.0000	0.0000	0.0000	0.0000	0.9527	0.0473	0.0000	0.0000	0.0119
Australia	0.0000	0.0000	0.0000	1.0000	0.0000	0.0000	0.0000	0.0000	0.0000	0.0000	0.0000	0.0000	0.0000	0.0000	0.0000	0.0000	0.0000	0.0000	0.0000	0.0902
Brazil	0.0000	0.0000	0.0000	0.0000	0.0000	0.0000	0.0000	0.0000	0.0000	0.0000	0.0000	0.0000	0.0000	0.0000	0.0000	0.0000	0.0000	0.0000	1.0000	0.0028
Canada	0.0000	1.0000	0.0000	0.0000	0.0000	0.0000	0.0000	0.0000	0.0000	0.0000	0.0000	0.0000	0.0000	0.0000	0.0000	0.0000	0.0000	0.0000	0.0000	0.1341
China	0.0000	0.0000	0.0000	0.0000	0.0000	0.0000	0.0000	0.0000	0.9398	0.0602	0.0000	0.0000	0.0000	0.0000	0.0000	0.0000	0.0000	0.0000	0.0000	0.0433
France	0.0000	0.0000	0.0000	0.0000	1.0000	0.0000	0.0000	0.0000	0.0000	0.0000	0.0000	0.0000	0.0000	0.0000	0.0000	0.0000	0.0000	0.0000	0.0000	0.0771
Germany	0.0000	0.0000	0.0000	0.0000	0.0000	1.0000	0.0000	0.0000	0.0000	0.0000	0.0000	0.0000	0.0000	0.0000	0.0000	0.0000	0.0000	0.0000	0.0000	0.0665
India	0.0000	0.0000	0.0000	0.0000	0.0000	0.0000	0.0000	0.0000	0.0000	0.0000	0.0000	0.0000	0.0000	0.0000	0.0000	0.0026	0.0981	0.8993	0.0000	0.0060
Indonesia	0.0000	0.0000	0.0000	0.0000	0.0000	0.0000	0.0000	0.0000	0.0000	0.0000	0.0000	0.0000	0.0000	0.9993	0.0007	0.0000	0.0000	0.0000	0.0000	0.0193
Italy	0.0000	0.0000	0.0000	0.0000	0.0000	0.0000	0.0000	0.0000	0.0000	0.1494	0.8506	0.0000	0.0000	0.0000	0.0000	0.0000	0.0000	0.0000	0.0000	0.0334
Japan	0.0000	0.0000	0.0000	0.0000	0.0000	0.0000	0.0000	1.0000	0.0000	0.0000	0.0000	0.0000	0.0000	0.0000	0.0000	0.0000	0.0000	0.0000	0.0000	0.0503
Mexico	0.0000	0.0000	0.0000	0.0000	0.0000	0.0000	0.0000	0.0000	0.0000	0.0000	0.0000	0.0000	0.0000	0.0007	0.9993	0.0000	0.0000	0.0000	0.0000	0.0156
Russia	0.0000	0.0000	0.0000	0.0000	0.0000	0.0000	0.0000	0.0000	0.0000	0.0000	0.0000	0.0000	0.0000	0.0000	0.0000	0.0447	0.8546	0.1007	0.0000	0.0086
Saudi Arabia	0.0000	0.0000	0.0000	0.0000	0.0000	0.0000	0.0000	0.0000	0.0602	0.7904	0.1494	0.0000	0.0000	0.0000	0.0000	0.0000	0.0000	0.0000	0.0000	0.0374
South Africa	0.0000	0.0000	0.0000	0.0000	0.0000	0.0000	0.0000	0.0000	0.0000	0.0000	0.0000	0.0000	1.0000	0.0000	0.0000	0.0000	0.0000	0.0000	0.0000	0.0234
South Korea	0.0000	0.0000	0.0000	0.0000	0.0000	0.0000	1.0000	0.0000	0.0000	0.0000	0.0000	0.0000	0.0000	0.0000	0.0000	0.0000	0.0000	0.0000	0.0000	0.0578
Turkey	0.0000	0.0000	0.0000	0.0000	0.0000	0.0000	0.0000	0.0000	0.0000	0.0000	0.0000	1.0000	0.0000	0.0000	0.0000	0.0000	0.0000	0.0000	0.0000	0.0278
United Kingdom	0.0000	0.0000	1.0000	0.0000	0.0000	0.0000	0.0000	0.0000	0.0000	0.0000	0.0000	0.0000	0.0000	0.0000	0.0000	0.0000	0.0000	0.0000	0.0000	0.1078
United States	1.0000	0.0000	0.0000	0.0000	0.0000	0.0000	0.0000	0.0000	0.0000	0.0000	0.0000	0.0000	0.0000	0.0000	0.0000	0.0000	0.0000	0.0000	0.0000	0.1867

**Fig 2:**
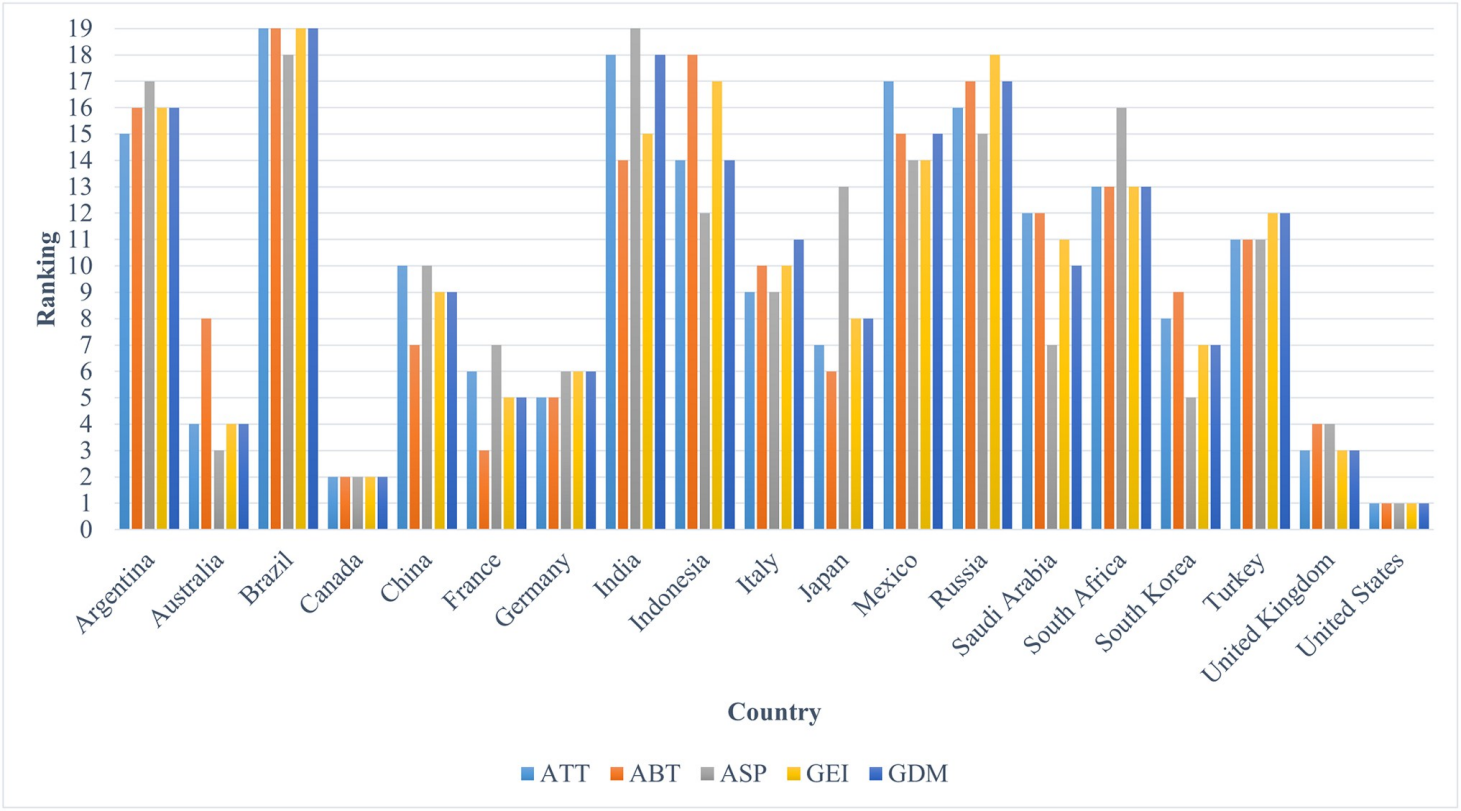
Ranking comparisons.

19 G20 countries are ranked in accordance with the obtained holistic acceptability indices, and are further compared with the ranking derived according to the GEI-2019. In addition, the rankings of ATT, ABT, and ASP are also reported for further comparisons, which are demonstrated in the Fig 2 below. We notice that 6 out of 19 countries are differently ranked between GEI and GDM, while the ranking positions of the rest countries are sufficiently robust and reliable. Additionally, most of the countries are ranked differently under the ATT, ABT, ASP, GEI and GDM, except for United States and Canada.

For the purpose of evaluating the effectiveness of our GDM method, we develop a satisfaction index to compare the degrees of satisfaction between the proposed GDM method ($a_{i}^{h}$) and individual decision maker. The difference between the holistic acceptability level of an alternative derived by our GDM method and that evaluated by a single decision maker could be a reasonable measure of satisfaction, that is,

$$\varphi_{ip}=\left|v_{ip}-a_{i}^{h}\right|$$ (18)

The above satisfactory difference is normalized as $\psi_{ip}^{NOR}=\frac{\varphi_{ip}}{\sum_{j=1}^{n}\varphi_{ip}}$, which has a conceptual interpretation by means of distance. In other words, the larger the satisfactory difference, the less satisfactory the alternative.

Aside from the satisfactory difference, the differences between the rankings derived in accordance with $a_{i}^{h}$ and any of other alternatives can also have a significant effect on the satisfactory levels of alternatives. Then the difference of preferential rankings is denoted as

$$\zeta_{ip}=\left|\rho_{ip}-\rho_{i}\right|$$ (19)

in which *ρ*_*ip*_ indicates the preferential ranking of alternative *i* assessed by DM *p*, and *p*_*i*_ represents the preferential ranking of alternative *i* in accordance with $a_{i}^{h}$.

In harmony with Siskos et al. [39], we therefore combine the above two satisfactory aspects to build the satisfaction index for DM *p* as below:

$$\kappa_{p}=1-\frac{1}{m}\sum_{i=1}^{m}\left[{\left(\psi_{ip}^{NOR}\right)}^{\zeta_{ip}}a_{i}^{h}\right]$$ (20)

According to the extant studies [7–9], the arithmetic average of *κ*_*p*_, *p* = 1, 2, …, 6 is utilized to represent the comprehensive satisfactory levels for all individual decision makers.

The simple additive weighting (SAW) method is usually recognized as common aggregation method for group decision, and is also known as the most widely-used method for real world group decision problems [9]. In this sense, the satisfaction indices of each decision maker are collected when using both SAW and our GDM methods, are reported in Table 6 below as well. We find that our GDM method could significantly increase the satisfactory levels of all decision makers. From the perspective of the decision group, the satisfactory level is improved as $\frac{0.9664-0.7234}{0.7234}\times 100\%=25.15\%$. Therefore, the proposed GDM method is more satisfactory than the SAW method in the group decision problem.

**Table 6:** Satisfaction index.

	SAW method	Proposed GDM method	Difference
TBS	0.6053	0.9581	+0.3528
TSB	0.7232	0.9602	+0.2370
BTS	0.7495	0.9705	+0.2210
BST	0.6374	0.9637	+0.3263
STB	0.7662	0.9686	+0.2024
SBT	0.8588	0.9773	+0.1186
Decision group	0.7234	0.9664	+0.2430

## 6. Conclusions

Complied and published by The GEDI Institute, the GEI is a composite indicator that measures the quality and dynamics of entrepreneurship ecosystems at a national and regional level across the 3As: ATT, ABT, and ASP. To deal with the drawbacks with respect to the arithmetic average aggregation scheme of the GEI, this study proposes to re-build the GEI by developing a GDM method with preference analysis. More specifically, an individual decision maker is firstly represented using a specified individual judgement about the importance order of various sub-indices of the GEI. A preliminary group decision matrix is therefore constructed by taking all possible individual judgments into account. The analysis about preferential differences and preferential priorities of the preliminary group decision matrix is the conducted to obtain a revised group decision matrix, in which preferential differences calculate the weighted differences as the degrees of differences among different alternatives for each decision maker, preferential priorities describe the favorite ranking of alternatives for each decision maker. We ultimately employ the Stochastic Multicriteria Acceptability Analysis for group decision-making (SMAA-2) to build the holistic acceptability indices for measuring the entrepreneurship performance. In addition, we create a satisfaction index to demonstrate the merits of proposed GDM method. An empirical study using the GEI-2019 of 19 G20 countries is carried out to show the implementation of our GDM method.

Our GDM method has several remarkable advantages over the previous measures. First, the proposed GDM method allows us to capture the collective wisdom from a group decision-making perspective. Second, the data-driven mechanism of our method reduces the decision bias and enhances the result objectiveness. Third, the preference analysis and aggregation views allow for more detailed understanding of the entrepreneurship performance. This would help inform policymakers in improving the entrepreneurship in a suitable manner.

Meaningful implications could be derived from this study. First, various individual preferences among ATT, ABT, and ASP should be taken into account when constructing the GEI, while the arithmetic average aggregation scheme has several endogenous shortcomings. Second, the investigation of preference structure about the group decision matrix would provide more practical decision supports. Third, the revised GEI is accepted by all individual decision makers. In other words, the disparity of individual preferences could be appropriately handled in this study.

There are some meaningful future research directions for this research. First, the effectiveness of individual decision makers’ decision results should be checked in the future research. For instance, the validity of closed-form solutions should be further verified. Second, apart from the preferential differences and preferential priorities for the group decision matrix, future research should investigate other aspects of preference analysis. Third, the merits of proposed GDM method should be further verified by performing more comparisons with more data or other aggregation methods in the future.

## Supporting information

S1 File (XLSX)
